# Bioprocess Optimization for the Production of *Arthrospira* (Spirulina) *platensis* Biomass Enriched in the Enzyme Alkaline Phosphatase

**DOI:** 10.3390/bioengineering8100142

**Published:** 2021-10-15

**Authors:** Giorgos Markou

**Affiliations:** Institute of Technology of Agricultural Products, Hellenic Agricultural Organization—Demeter, L. Sof. Venizelou 1, 14123 Lykovrysi, Greece; gmarkou@itap.com.gr

**Keywords:** alkaline phosphatase, Spirulina, functional food, bioprocess, enzymes

## Abstract

The enzyme alkaline phosphatase (ALP) is gaining interest because it exerts bioactive properties and may be a potentially important therapeutic agent for many disorders and diseases. Microalgae are considered an important novel source for the production of diverse bio-compounds and are gaining momentum as functional foods/feeds supplements. So far, studies for the production of ALP are limited to mammalian and partly to some heterotrophic microbial sources after its extraction and/or purification. **Methods:** *Arthrospira* was cultivated under P-limitation bioprocess and the effect of the P-limitation degree on the ALP enrichment was studied. The aim of this work was to optimize the cultivation of the edible and generally-recognized-as-safe (GRAS) cyanobacterium *Arthrospira platensis* for the production of single-cell (SC) biomass enriched in ALP as a potential novel functional diet supplement. **Results:** The results revealed that the relationship between intracellular-P and single-cell alkaline phosphatase (SC-ALP) activity was inverse; SC-ALP activity was the highest (around 50 U g^−1^) when intracellular-P was the lowest possible (around 1.7 mg-P g^−1^) and decreased gradually as P availability increased reaching around 0.5 U g^−1^ in the control cultures. Under the strongest P-limited conditions, a more than 100-fold increase in SC-ALP activity was obtained; however, protein content of *A. platensis* decreased significantly (around 22–23% from 58%). Under a moderate P-limitation degree (at intracellular-P of 3.6 mg-P g^−1^), there was a relatively high SC-ALP activity (>28 U g^−1^) while simultaneously, a relative high protein content (46%) was attained, which reflects the possibility to produce *A. platensis* enriched in ALP retaining though its nutritional value as a protein rich biomass source. The paper presents also results on how several parameters of the ALP activity assay, such as pH, temperature etc., and post-harvest treatment (hydrothermal treatment and biomass drying), influence the SC-ALP activity.

## 1. Introduction

Phosphatases belong to the superfamily of metalloenzymes that hydrolyze a variety of organic phosphorus compounds into orthophosphate. Among the different phosphatases, ALP (EC 3.1.3.1), a non-specific phosphomonoesterase [1], is gaining interest because of its potential for diverse applications in analytical chemistry, environmental engineering, food/feed production, and medicine. More specifically, ALP could be used as a labeling enzyme applied in electrochemical immunoassays and immunosensors [2], or applied in environmental engineering systems for the biodegradation of organophosphorus pesticides [3]. Moreover, ALP exerts anti-inflammatory properties and may be a potentially important therapeutic agent for many disorders and diseases [4,5,6,7]. For example, Martínez-Moya et al. [4] showed that the administration of exogenous ALP complemented endogenous enzyme protection in colonic inflammation and reduced bacterial translocation in rats. More recently, Kühn et al. [8] have shown that oral administration of ALP promotes the growth of intestinal symbiotic bacteria conserving homeostasis of the gut microbiota protecting intestine from inflammation caused by bacterially derived proinflammatory factors. They concluded that oral ALP supplementation might be a novel therapy against chronic inflammation related to aging process in humans. In another study, Haarhaus et al. [5] suggest that ALP could act as a novel treatment against cardiovascular disease, a main cause of early death in the settings of chronic kidney disease. Other potential biomedical uses of ALP include bone tissue engineering [9].

Microalgae (including cyanobacteria) are becoming a very promising source of nutritional food/feed, and nowadays, some species, like *Arthrospira platensis* (commonly known as Spirulina) and *Chlorella vulgaris*, are commercially cultivated to produce biomass traded as food supplements [10]. Moreover, microalgae are gaining momentum as a novel source for the development and production of functional foods/feeds. This is because they synthesize various bioactive metabolites such as polysaccharides [11,12], polyunsaturated fatty acids [13,14], antioxidant compounds [15,16], vitamins [17,18], etc., that have proven health promoting properties, and therefore, they could be used as ingredients to develop novel functional foods/feeds [19,20,21,22,23,24]. Besides these nutritional compounds, there is also a huge potential to produce diverse intracellular or extracellular high-value products from microalgae including enzymes, such as cellulases, proteases, galactosidases, lipases, phosphatases, etc., which might have diverse applications in food or chemistry sectors [25,26,27].

The sources studied so far for potential ALP production are limited only to mammalian and partly to some heterotrophic microbes [3,28,29]. A major disadvantage facing these production sources is that they necessitate the extraction and/or purification of ALP in order to be used, especially in food/feed applications. Since *A. platensis* is one of the few microalgae species considered so far to be generally-recognized-as-safe (GRAS) and its consumption is permitted, the production of microalgal SC-ALP might be of particular interest as a simpler means to orally administrate ALP as healthy food/feed supplement. *Arthrospira* species exert also important biomedical properties such as anticancer, antihypertensive, and anti-inflammatory activities and immune system enhancement [30]. It was also found that it exerts nutritional properties such as anti-diabetes and anti-obesity activities, while it could be used as an agent against heavy metal toxicity or as anti-microbial agent; *Arthrospira* contains a relative high content of antioxidants, vitamins, and trace elements and therefore belongs to the category of superfoods with great potential to be used as ingredient for the development of functional foods/feed [30,31,32]. *A. platensis* has been successfully incorporated in various food products, such as cookies [33], bread [34], pasta [35], cheese [36], etc., or in feed for feeding fish [37], poultry [38], piglets [39], etc. Moreover, *A. platensis* is a photosynthetic and alkalophilic microorganism which might provide easier cultivation facilities (no sterilization needed, low contamination potentials, etc.) for the production of SC-ALP.

So far, the studies dedicated to microalgal ALP are focused mainly on ALP extraction, purification and characterization processes [27], while there is in general a lack of data on the production process and optimization of microalgal SC-ALP and on some downstream treatment parameters (such as drying of biomass) that might affect the SC-ALP activity. Thus, the present study aimed to bring to the fore and provide some aspects on this potentially new topic of microalgal SC biomass enriched in enzymes. This report will present its results in three main sections: (i) how the cultivation process, in particular different phosphorus limitation degrees, affects the biomass production and the SC-ALP content in order to optimize the SC-ALP production; (ii) how several parameters of the ALP activity assay, namely pH, incubation temperature, the addition of chelator (EDTA), and the addition of metal cations (Ca^2+^. Mg^2+^ and Zn^2+^) influence the SC-ALP activity; and (iii) how downstreaming, i.e., post-harvest treatments, such as hydrothermal thermal treatment, and thermal biomass drying, affect the thermostability and SC-ALP activity, respectively.

## 2. Materials and Methods

### 2.1. Microorganism and Cultivation Conditions

The cyanobacterium *Arthrospira platensis* SAG 21.99 used in the study was obtained from SAG (Sammlung von Algenkulturen der Universität Göttingen). *A. platensis* was grown in modified Zarrouk medium with the following composition (per liter): 16.8 g NaHCO_3_, 1.0 g K_2_SO_4_, 1.0 g NaCl, 0.04 g CaCl_2_, 0.08 g Na_2_EDTA, 0.2 g MgSO_4_·7H_2_O, 0.01 g FeSO_4_·7H_2_O and 1.0 mL of trace elements (per l): 2.86 g H_3_BO_3_, 0.02 g (NH_4_)_6_Mo_7_O_24_, 1.8 g MnCl_2_·4H_2_O, 0.08 g CuSO_4_·5H_2_0 and 0.22 g ZnSO_4_·7H_2_O, while phosphorus (supplied in form of K_2_HPO_4_) was provided in different concentrations (see Section 2.2 for more details). Cultivations were carried out in closed cylindrical glass photobioreactors with an inner diameter of 76 mm and working volume of 0.5 l. The cultures were aerated with filtered air provided by a membrane air pump for agitation reasons. Cultivation was performed in a room with controlled temperature at 28 °C (± 2 °C). Light intensity was set at 150 μmol m^−2^ s^−1^ (measured by SpectraPen, PSI, Czech Republic) and was provided continuously through a 30W LED panel on the one side of the photobioreactors.

### 2.2. Experimental Design

The cultures were performed in aa semi-continuous mode and lasted for more than 2.5 months in order to ensure a steady state and reliable results. The dilution rate was set at 20% and feeding performed every day, 7 days per week. All nutrients, including phosphorus, were fed semi-continuously at a volume equal to the volume withdrawn from the cultures according to the dilution rate (20%). The phosphorus was supplied in the range of 0.75–9 mg L^−1^ (0.75, 1, 2, 3, 4.5, 6, 7.5 and 9 mg L^−1^), while the control culture contained about 89 mg L^−1^ (0.5 g L^−1^ K_2_HPO_4_). In all cultures (except that of the control), phosphorus measured in the daily withdrawn portion of the growth medium was completely removed (less than the detection limit of 50 μg-P L^−1^), and it was assumed that all of the provided phosphorus was taken up by *A. platensis* and therefore it was considered that the removed P was transformed into intracellular-P (mg-P per g of DW biomass). The main aim of the cultivation design was to investigate if there is any relationship between intracellular-P (as an indicator of the degree of P-limitation level) and ALP enrichment of biomass, and subsequently to produce biomass for SC-ALP activity studies.

Besides the main experimental series, another triplicate of cultures with 1.5 mg-P L^−1^ were subjected in lower light intensity (PAR of 50 μmol m^−2^ s^−1^) in order to study whether light conditions have any effect on the SC-ALP production capacity of *A. platensis*. In another series of cultures, nitrogen and potassium were also completely omitted from the growth medium in order to perceive if there was any effect on the ALP biosynthesis in *Arthrospira* cultivated under P replete conditions. All cultures were carried out in triplicates and results are their mean values (±standard deviation).

### 2.3. Analytical Methods

Dry algal biomass was measured after harvesting the cells of an aliquot of 50 mL in a paper filter (40 μm pore size) and drying it in an oven at 80 °C for 3 h. For the various analyses and assays, the cells were harvested using centrifugation (5000 rpm for 5–10 min) and washed at least 3 times with DI water in order to exclude the salts of the growing medium. Proteins, carbohydrates, lipids, phycocyanin and phosphorus were measured according to the analytical methods used in a previous study [40]. All above colorimetric analyses were performed using a spectrophotometer (Cadas 50, Dr. Lange, Germany).

ALP activity was assayed with the p-nitrophenyl phosphate (p-NPP) method [41,42]. Briefly, a 250 μL sample of fresh biomass, 125 μL of buffer solution and 250 μL of p-NPP were mixed and incubated for 20 min in an incubator with controlled temperature. Τhe reaction was stopped by the addition of 125 μL of 4N NaOH and the samples where centrifuged for 5 min. The optical absorption of the released p-nitrophenyl in the supernatant of the reaction mixture was measured at 410nm in a microplate reader (Molecular Devices, SpectraMax 340PC, San Jose, CA, USA). For the calibration curve, p-nitrophenyl was used. The specific ALP activity was calculated as units per 1 g of biomass (DW, Bonn, Germany) of *A. platensis*. One unit of ALP activity was defined as the quantity of the enzyme required to release 1 μmol of p-NPP per min [41]. All assays for ALP activity were performed using fresh biomass that was withdrawn every day from the cultures. Biomass was separated through centrifugation and washed 3 times and resuspended in DI water. Τhe standard SC-ALP activity assays were performed using a 1M buffer of pH 12.5 (KCl/NaOH), 2.5 μM p-NPP and temperature of 37 °C. In any other case, the different assay conditions will be stated in the text. All analyses were carried out at least in triplicates for each replicate; however, the results given are the average of the three replicates (n = 3) ± standard deviation (SD). For the calculation of the Michaelis–Menten constant (K_m_; mM) and the reaction velocity (V_max_; mM g_biomass_^−1^ min^−1^), SC-ALP was assayed by ranging p-NPP concentration from 40 to 0.0375 mM. Data were linearized through Lineweaver–Burk transformation.

### 2.4. Statistical Analysis

Statistical analysis was performed using SigmaPlot v.12.0 (Systat Software, Inc., San Jose, CA, USA). Before conducting the statistical analysis, the data were checked for normality and homogeneity by the Shapiro–Wilks test. To test the statistical differences, a one-way ANOVA test with Duncan post-hoc was performed. The data were displayed as means ± standard deviation (SD) of three biological (independent) replicates (n = 3). For each biological replicate, a triplicate of analyses were performed. Data were considered significantly different at *p* ≤ 0.05.

## 3. Results and Discussion

### 3.1. Phosphorus Concentration Effect on Biomass Production and Biochemical Composition

**Figure 1:**
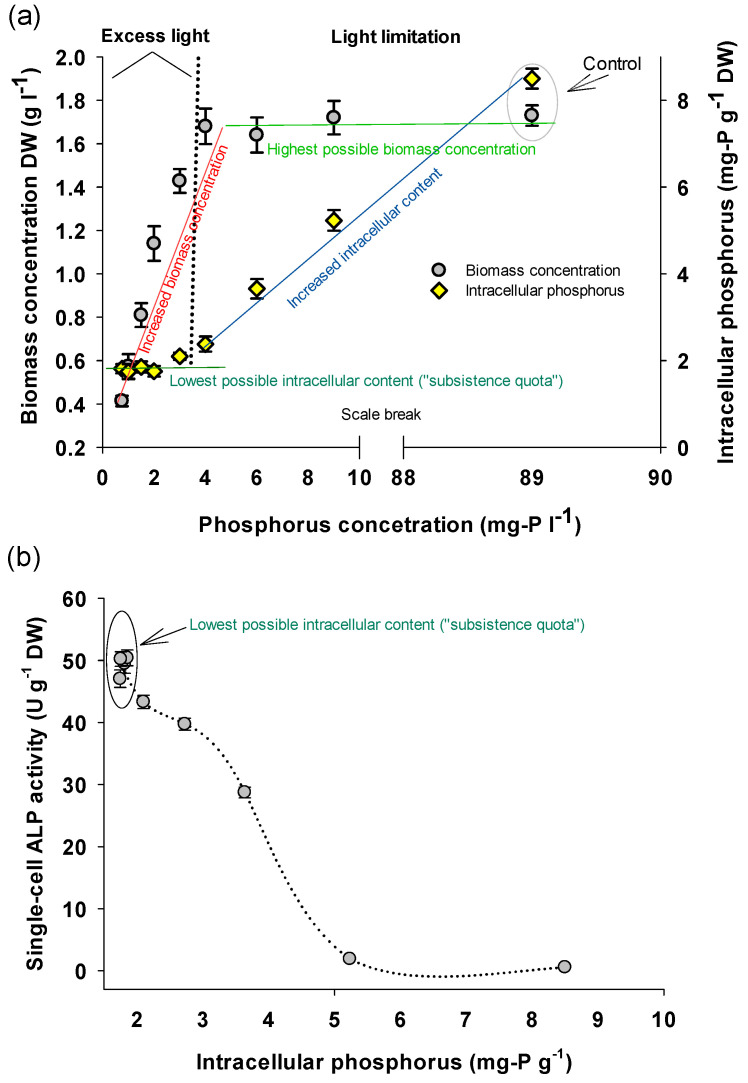
(**a**) Relationship between phosphorus concentration with biomass production and intracellular phosphorus content of *A. platensis* cultivated in semi-continuous mode with diverse phosphorus limitation degrees. (**b**) Relationship between intracellular phosphorus content and single-cell alkaline phosphatase activity of *A. platensis*.

Figure 1 illustrates the biomass concentration of *A. platensis* cultivated in semi-continuous mode with different concentrations of phosphorus. The cultures were kept for more than 2.5 months of operation and the biomass concentrations shown in Figure 1a were in effect unchanged for more than 1.5 months, where during this period, all analytical work was performed using freshly harvested biomass.

As shown in Figure 1a, in the low end of P concentrations (0.75–4 mg-P L^−1^), there was a stepwise increase (*p* < 0.05) of biomass production as P increased up to 3 mg-P 4^−1^. This biomass increase was proportional to P provided, i.e., of about 150 mg DW for every additional 250 μg-P 4^−1^. By increasing the concentration from 4 mg-P l^−1^ and further, the biomass concentration reached a plateau (*p* > 0.05) ranging between 1.64–1.73 g 4^−1^. In contrast, intracellular-P was in its lowest level (around 1.7–2.1 mg-P g^−1^) in the cultures with the lowest P concentration (of up to 3 mg-P L^−1^), while it increased gradually when P provided in higher amounts. At this point, it was assumed that the cultures with up to 3 mg-P L^−1^ were not light-limited and the biomass production capacity was ruled by the available P, which should have been the main growth limiting factor. This is based on the fact that there is a threshold of an intracellular nutrient content, the so-called ‘‘subsistence quota”, where when the intracellular nutrient content reaches this threshold, algae do not grow further [43]. When nutrients are replete, other environmental and cultivation limiting factors, such as light, exist, which determine the overall maximum biomass production potential of a specific cultivation system.

The cultures in this study became light limited at higher P concentrations (>4 mg-P L^−1^) where biomass concentration reached that given plateau, where no further biomass could be produced due to the cell-shading effect [44]. The amount of P provided and subsequently the intracellular-P affected strongly the biochemical composition of *A. platensis* as shown in Table 1; at the lower P concentrations, the higher SC-ALP content was accompanied with higher carbohydrates, and lower protein and phycocyanin content. In other words, there is a trade-off between accumulation of ALP and protein and phycocyanin content.

ALP content (expressed as SC-ALP activity U g^−1^) was significantly impacted by the P availability (Figure 1b); more specifically the relationship between intracellular-P and SC-ALP activity was inverse. The highest values of ALP activity (around 50 U g^−1^ of DW biomass) were obtained with the lowest intracellular-P (at ‘‘subsistence quota” level), while increasing intracellular-P, SC-ALP activity decreased reaching the lowest value of around 1.7 U g^−1^ of DW biomass in the control cultures. It is hypothesized here that the increased SC-ALP activity reflects the intensification of the efforts that cells make to cope with decreased P-availability by increasing the probability to gain P from the surroundings by hydrolyzing compounds that might contain P. These results come in general in agreement with previous studies of Markou [40] and Adams et al. [45], where it was shown that a specific relationship between intracellular nutrient limitation and biomass composition (for example carbohydrates and lipids) of several microalgal species exists. Explicitly, at a specific minimum intracellular nutrient concentration, the desired biomass compound content is accumulated gradually as intracellular nutrient concentration approaches the ‘‘subsistence quota” level.

The above conclusion is confirmed also by the additional experimental series conducted where *A. platensis* was cultivated with phosphorus concentration of 1.5 mg-P L^−1^ but at lower light intensity (50 μmol m^−2^ s^−1^ photon flux). The biomass produced in this experimental series had intracellular-P of 3.8 mg-P g^−1^ and SC-ALP activity of around 25 U g^−1^. This result suggests that light had an effect on SC-ALP activity only because it regulates the biomass production capacity of the culture, and thus the intracellular-P content for given P concentration provided, which in turn regulates the SC-ALP content (expressed as SC-ALP activity).

Interestingly, *A. platensis* with an intracellular-P of 3.6 mg-P g^−1^ had a relatively high ALP activity (>28 U g^−1^) and at the same time, a relatively high protein content (46%; Table 1). This shows that it is possible that under optimized conditions, *A. platensis* could be produced as SC protein enriched in ALP, i.e., combining the nutritional value of *A. platensis* proteins with the potential functionality of the biomass because of the enrichment in ALP.

In order to investigate whether ALP activity could be increased by other nutrient limitation, nitrogen and potassium depleted conditions were also studied. The results (data not shown) showed that there was no SC-ALP activity increase compared to the control (replete P cultures), which suggest that ALP is not synthesized due to nitrogen or potassium starvation. Although microalgal ALP activity has been extensively investigated in studies on the field of ecology [46,47], to the best knowledge of the author, this is the first report on how the intracellular-P content affects the SC-ALP activity providing a deeper insight for the optimization of SC-ALP production by the photosynthetic cyanobacterium *A. platensis*.

ALP production by microalgae has been extensively studied as an indicator of the trophic conditions (P availability) of natural environments concerning the ecological and environmental significance of eutrophication. It is known that different classes of microbes (bacteria, eukaryotic microalgae, cyanobacteria, etc.) synthesize ALP as a mechanism to cope with low P availability [48,49], a fact that is confirmed also in the present study. Nevertheless, there is lack of studies dealing with the bioprocess optimization of ALP production by microalgae and cyanobacteria, where the present work aims to give some new perspectives on the exploitation of ALP production. Studies towards production optimization of ALP with the aim to be used in different applications focus mainly on heterotrophic microbes that excrete ALP in the surroundings which is then recovered and purified [50,51]. Moreover, there is a strong diversity between various microbial species and different genes (regulons) are responsible for the synthesis of ALP isozymes [52]. The amino-acid sequences of the enzymes impact their activity, where some sources like mammalian cells give more active (20–30-fold) ALP than other bacterial ones [53]. Consequently, there is more research needed to better understand the differences that occur between the various sources. Since ALP produced by *A. platensis* is homologous to the ALP types of the other sources, it is hypothesized that the SC-ALP could find applications in developing of medical or functional foods/feed. For example SC-ALP could be applied as an ingredient in fish feed as an agent to compact the toxicity derived from contaminated waters with organophosphorus pesticides, such as Chlorpyrifos [24].

### 3.2. Parameters Affecting the Enzymatic Activity of Single-Cell Alkaline Phosphatase

**Figure 2:**
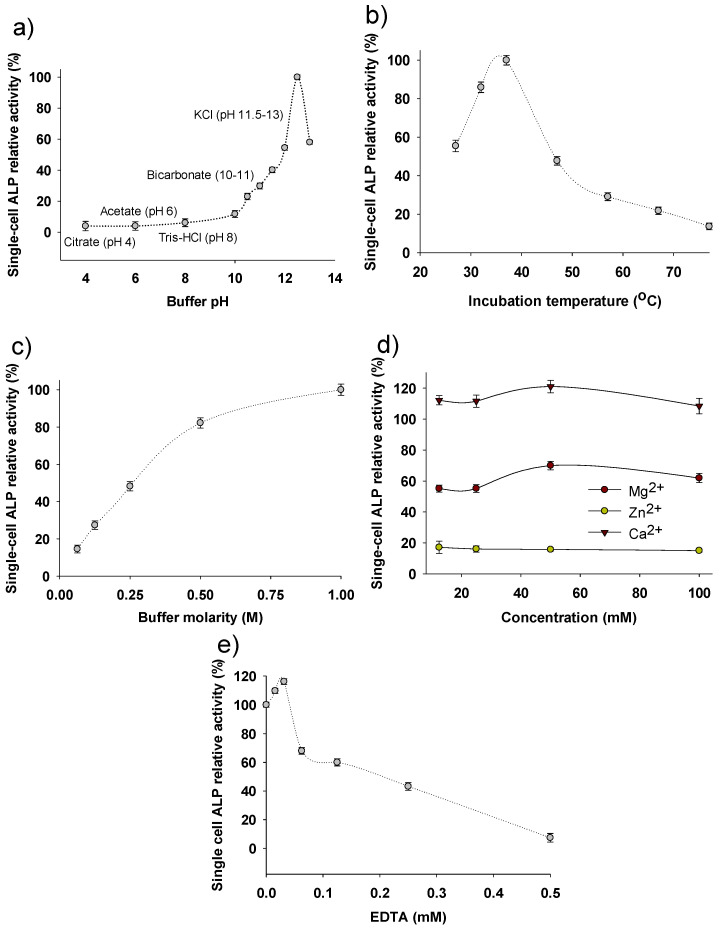
Effect of selected parameters on the single-cell alkaline phosphatase activity assay: (**a**) effect of the pH of the buffer (1 M KCl + NaOH, pH 12.5, T = 37 °C), (**b**) temperature of incubation (1 M KCl, pH 12.5), (**c**) molarity of the buffer (pH 12.5, T = 37 °C), (**d**) of the treatment of biomass with metal ions (Mg^2+^, Zn^2+^, and Ca^2+^); (1 M KCl, pH 12.5, T = 37 °C) and (**e**) of the treatment of biomass with EDTA (1 M KCl, pH 12.5, T = 37 °C). Data shown are the mean ± SD of n = 3.

In this section, the main parameters of the SC-ALP activity assay are investigated in order to partially characterize the *A. platensis* SC-ALP. The effect of buffer pH (Figure 2a), incubation temperature (Figure 2b) and buffer molarity (Figure 2c) on the SC-ALP activity is shown. The SC-ALP activity was increased with increasing pH displaying a sharp peak at pH 12.5. *A. platensis* is an alkalophilic cyanobacterium that thrives in growing media rich in bicarbonates/carbonates and pH values greater than 9.5 (in the present study, the pH of the growth media ranged between 10.00 and 10.50), a fact that explains the affinity of this ALP for higher pH values. The value of pH 12.5 found in this study is higher than it was observed in extracted and purified ALP on the works of Thengodkar and Sivakami [54] and Asencio et al. [55], who both reported optimum pH of 11.5 (*p* < 0.05); however, it is worth noting that pH 11.5 was the highest value assayed in both the studies of Thengodkar and Sivakami [54] and Asencio et al. [55] and it is probable that they would have found higher pH values if they had assayed a wider pH range. The best temperature for the ALP activity, as shown in Figure 2b, was 37 °C (*p* < 0.05), with a steep decrease when the temperature raised to 47 °C and above. Similar results were reported also by Singh et al. [56] in the cyanobacterium *Anabaena oryzae* where SC-ALP activity was the highest at temperature around 35–38 °C. Regarding the effect of the buffer (KCl + NaOH) molarity on the SC-ALP activity (Figure 2c), the highest ALP activity measured was obtained in 1M buffer, where at the half of the molarity (0.5 M), ALP activity was around 80% (and statistically different, *p* < 0.05) of that of 1M. In the study of Upadhyay and Verma [57], where extracted ALP from milk was assayed, it was reported that the optimum ionic strength (molarity) of the buffer was 0.5M, while increasing it further, there was a slight decrease in ALP activity up to 0.8M of buffer. However, these differences could be due to the fact that SC-ALP is located either in the cell wall or in the inner layer of the sheath of the filament of *A. platensis* [55] which might require stronger buffer for enhanced activity or that the polysaccharides of the cell walls act as protective agents against higher ionic strengths.

In Figure 2d,e, the effect of the addition of metal cations (Mg^2+^, Zn^2+^ and Ca^2+^), and of the chelator (EDTA), respectively, on the SC-ALP activity is shown. SC biomass was incubated at room temperature for 2 h in the presence of the cations and was then centrifuged and washed 2 times with DI water. The addition of Mg^2+^ and Zn^2+^ resulted in a decreased SC-ALP activity, displaying around 55–70% and 15–17% of the standard activity, reflecting the inhibitory effect of these metal ions on the SC-ALP activity. In contrast, Ca^2+^ resulted in significantly higher values (108–121% of the standard activity), which reflects that SC-ALP activity was enhanced by the addition of calcium. These findings are in line with those of Asencio et al. [55], who concluded that ALP of *A. platensis* is calcium-depended and requires this metal ion to be active. Regarding the addition of EDTA, at very low concentrations (0.015–0.03 mM), there was an increase (110–116% of the standard activity) in the SC-ALP activity, while at higher concentrations, the activity strongly decreased. At low doses of EDTA, it is presumed that the increased activity is due to the chelation and removal of some inhibitory metals (probably trace elements originating from the growth medium) that were attached to the enzymes, while at higher doses, EDTA might cause chelation of the required metal enzyme co-factor (such as calcium) resulting in apoenzymes, and therefore, in inactivation of the enzymes [55,58].

**Figure 3:**
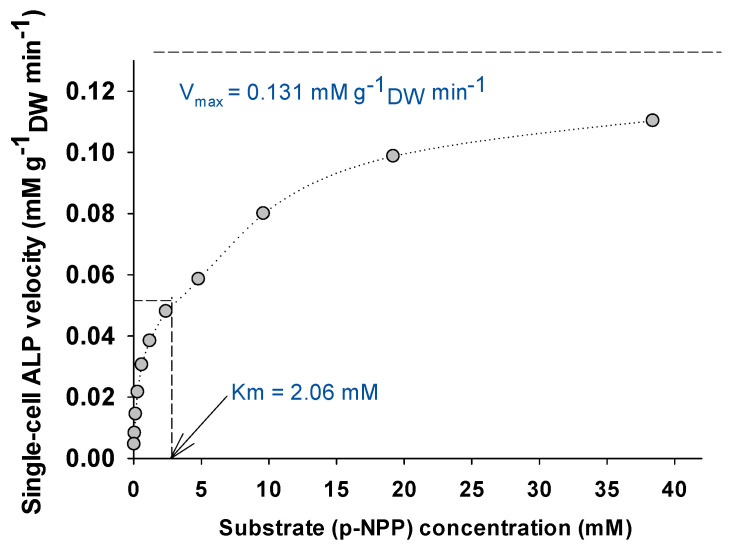
Michaelis–Menten kinetics of *A. platensis* single-cell enriched in alkaline phosphatase (standard assay conditions: 1 M KCl, pH 12.5, T = 37 °C).

Figure 3 shows the Michaelis–Menten kinetics of SC-ALP activity by varying the substrate (p-nitrophenyl phosphate; p-NPP) concentration. Under standard assay conditions, and after linearization of the data through the Lineweaver–Burk transformation (R^2^ = 0.9955), the Michaelis–Menten constant K_m_ and the maximum reaction rate V_max_ of the SC-ALP of *A. platensis* was calculated as 2.06 mM and 0.131 mM g_biomass_^−1^ min^−^^1^, respectively. K_m_ gives an estimation of the substrate’s affinity for the enzyme, where small K_m_ indicates higher affinities. The K_m_ of ALP varied greatly and can range between 0.011 and 15 mM, depending on its origin (microbial or mammalian origin) and the activity assay conditions [59]. There are no available published data on the K_m_ of ALP from *A. platensis* or only few from other microalgae in order to compare the results of the present study. It is hypothesized that the K_m_ of enzymes associated attached in the biomass will display higher values, i.e., lower affinities, since the cell wall itself could be a kind of barrier for the maximum activity of the enzymes. This hypothesis could be confirmed by the K_m_ of the extracellular and purified ALP from the microalga *Chlamydomonas reinhardtii*, which was 0.517 mM [41]. Nevertheless, more research is needed in order to have a deeper inside on the SC-enzyme kinetics.

### 3.3. Downstream Treatments Affecting the Single-Cell ALP Activity

**Figure 4:**
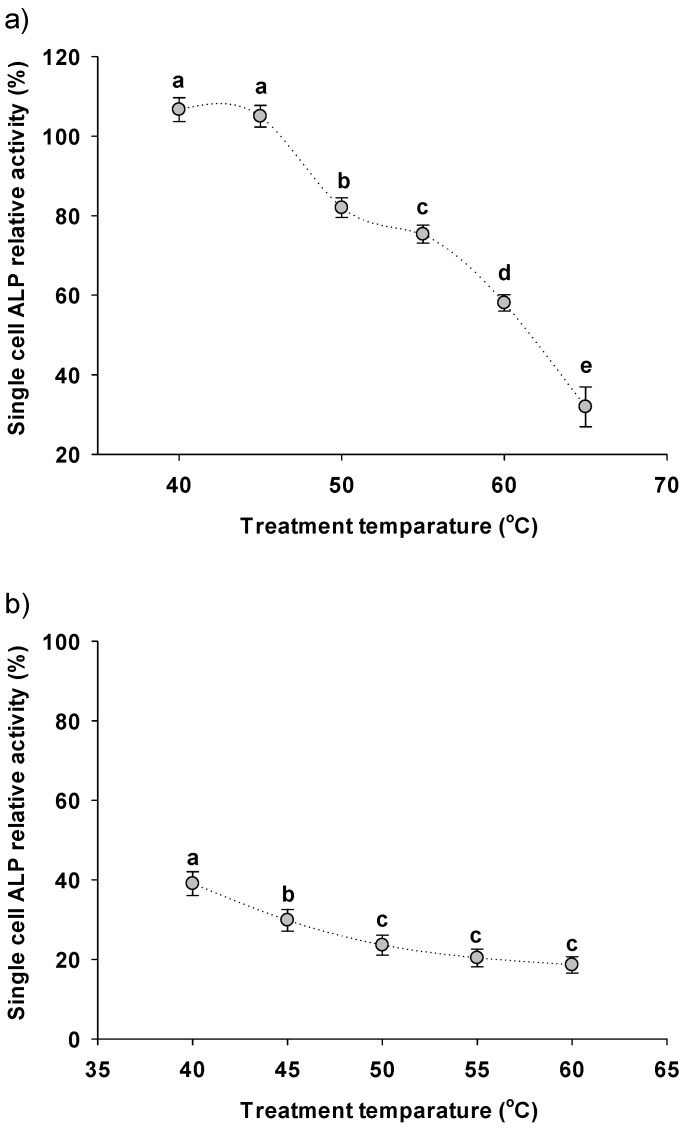
Effect of (**a**) hydrothermal treatment and (**b**) drying temperature of single-cell ALP activity of *A. platensis* enriched in ALP. Data shown are the mean ± SD of n = 3. Same alphabetical symbol denoted no statistically significant differences between the means.

Figure 4a illustrates the SC-ALP activity of *A. platensis* after its hydrothermal treatment for 2 h under different temperatures (40–65 °C). As shown there, when *A. platensis* was treated with 40 and 45 °C, it displayed a slight increase in SC-ALP activity (around 105% of the standard SC-ALP activity), while at higher temperatures, it was decreased, gradually reaching around 25% of the SC-ALP activity at 65 °C. The results are in total agreement with Thengodkar and Sivakami [54], who also observed that extracted ALP activity from *A. platensis* had a gradual decrease as temperature treatment increased from 50 to 80 °C. In addition, in this study, *A. platensis* enriched in ALP was subjected to a biomass drying process in order to investigate the effect of drying on the SC-ALP activity. As shown in Figure 4b, SC-ALP activity was strongly affected by drying even at the lower temperature (40 °C), displaying almost 40% of the initial SC-ALP activity, and decreased to around 20% upon increasing temperature.

It is hypothesized here that the drying and dewatering per se of biomass is a stronger factor that affects SC-ALP activity compared to the temperature. This is based on three observations: (i) the SC-ALP activity of hydrothermal treated *A. platensis* was in general higher than the dried biomass, (ii) at 40–45 °C, the SC-ALP of the hydrothermal treated *A. platensis* was even enhanced in contrast to the dried biomass where SC-ALP activity was significantly decreased, showing that the temperature per se is not an inactivation factor, and (iii) the curve of the SC-ALP activity of the hydrothermal treatment (Figure 4a) was steeper compared to the drying process (Figure 4b), suggesting that the dewatering of the biomass was a stronger inactivation factor than temperature. The effect of drying in the activity of different enzymes in plant materials are previously also observed [60].

## 4. Conclusions

SC-ALP activity was proportional to the intracellular-P content, which is the main parameter that influences the biomass ALP content. Under optimized conditions, it is possible to produce *A. platensis* with relative high protein content (46%), while at the same time, to be enriched in ALP (28 U g^−1^). SC-ALP displayed optimum activity at pH 12.5, temperature 37℃ and buffer molarity 1M. The addition of Ca^2+^ enhanced the SC-ALP activity. Drying of the biomass strongly decreased the SC-ALP activity; the dewatering of biomass was a stronger factor than the temperature of drying.

## Figures and Tables

**Table 1:** Biochemical composition of *A. platensis* cultivated in semi-continuous mode with different phosphorus concentrations. Data shown are the mean ± SD of n = 3.

P concentration (mg-P L^−1^)	Intracellular P (mg-P g^−1^)	Proteins %_DW_)	Carbohydrates (%_DW_)	Phycocyanin (%_DW_)	Lipids (%_DW_)
0.75	1.81	23.55 ± 2.37 ^a^	65.66 ± 7.55 ^a^	1.52 ± 0.23 ^a^	6.53 ± 1.23 ^abc^
1	1.74	22.67 ± 1.65 ^a^	64.24 ± 3.44 ^a^	1.49 ± 0.32 ^ab^	6.14 ± 0.69 ^ac^
1.5	1.85	23.88 ± 2.14 ^a^	66.12 ± 2.78 ^a^	1.67± 0.12 ^a^	5.98 ± 1.11 ^ac^
2	1.75	22.44 ± 2.98 ^a^	65.46 ± 6.79 ^a^	1.22 ± 0.23 ^b^	6.99 ± 1.36 ^abc^
3	2.10	25.39 ± 2.44 ^a^	62.99 ± 4.22 ^a^	2.45 ± 0.55 ^c^	8.45 ± 0.96 ^b^
4	2.73	31.49 ± 1.94 ^b^	42.14 ± 3.47 ^b^	7.78 ± 1.05 ^d^	5.44 ± 0.55 ^c^
6	3.64	45.96 ± 3.59 ^c^	25.83 ± 2.59 ^c^	9.98 ± 0.98 ^e^	7.76 ± 2.01 ^ab^
9	5.23	55.58 ± 4.17 ^d^	14.29 ± 1.55 ^d^	12.32 ± 2.04 ^f^	7.42 ± 1.87 ^ab^
89 (Control)	8.55	58.48 ± 3.42 ^d^	12.11 ± 2.84 ^d^	12.12 ± 1.26 ^f^	7.29 ± 1.84^ab^

Same alphabetical symbol denoted no statistically significant differences.

## References

[1] Green M.R., Sambrook J. (2020). Alkaline Phosphatase. Cold Spring Harb. Protoc..

[2] Zhao D., Li J., Peng C., Zhu S., Sun J., Yang X. (2019). Fluorescence Immunoassay Based on the Alkaline Phosphatase Triggered In Situ Fluorogenic Reaction of o-Phenylenediamine and Ascorbic Acid. Anal. Chem..

[3] Meng D., Jiang W., Li J., Huang L., Zhai L., Zhang L., Guan Z., Cai Y., Liao X. (2019). An Alkaline Phosphatase from *Bacillus amyloliquefaciens* YP6 of New Application in Biodegradation of Five Broad-Spectrum Organophosphorus Pesticides. J. Environ. Sci. Health Part B.

[4] Martínez-Moya P., Ortega-González M., González R., Anzola A., Ocón B., Hernández-Chirlaque C., López-Posadas R., Suárez M.D., Zarzuelo A., Martínez-Augustin O. (2012). Exogenous Alkaline Phosphatase Treatment Complements Endogenous Enzyme Protection in Colonic Inflammation and Reduces Bacterial Translocation in Rats. Pharmacol. Res..

[5] Haarhaus M., Brandenburg V., Kalantar-Zadeh K., Stenvinkel P., Magnusson P. (2017). Alkaline Phosphatase: A Novel Treatment Target for Cardiovascular Disease in CKD. Nat. Rev. Nephrol..

[6] Fawley J., Gourlay D.M. (2016). Intestinal Alkaline Phosphatase: A Summary of its Role in Clinical Disease. J. Surg. Res..

[7] Lallès J.-P. (2019). Recent Advances in Intestinal Alkaline Phosphatase, Inflammation, and Nutrition. Nutr. Rev..

[8] Kühn F., Adiliaghdam F., Cavallaro P.M., Hamarneh S.R., Tsurumi A., Hoda R.S., Munoz A.R., Dhole Y., Ramirez J.M., Liu E. (2020). Intestinal Alkaline Phosphatase Targets the Gut Barrier to Prevent Aging. JCI Insight.

[9] Osathanon T., Giachelli C.M., Somerman M.J. (2009). Immobilization of Alkaline Phosphatase on Microporous Nanofibrous Fibrin Scaffolds for Bone Tissue Engineering. Biomaterials.

[10] Torres-Tiji Y., Fields F.J., Mayfield S.P. (2020). Microalgae as a Future Food Source. Biotechnol. Adv..

[11] Markou G., Eliopoulos C., Argyri A., Arapoglou D. (2021). Production of *Arthrospira* (Spirulina) *platensis* Enriched in β-Glucans through Phosphorus Limitation. Appl. Sci..

[12] Bernaerts T.M., Gheysen L., Kyomugasho C., Kermani Z.J., Vandionant S., Foubert I., Hendrickx M.E., Van Loey A.M. (2018). Comparison of Microalgal Biomasses as Functional Food Ingredients: Focus on the Composition of Cell Wall Related Polysaccharides. Algal Res..

[13] Morales-Sánchez D., Schulze P.S., Kiron V., Wijffels R.H. (2020). Production of Carbohydrates, Lipids and Polyunsaturated Fatty Acids (PUFA) by the Polar Marine Microalga *Chlamydomonas malina* RCC2488. Algal Res..

[14] Barta D.G., Coman V., Vodnar D.C. (2021). Microalgae as Sources of Omega-3 Polyunsaturated Fatty Acids: Biotechnological Aspects. Algal Res..

[15] Sansone C., Brunet C. (2019). Promises and Challenges of Microalgal Antioxidant Production. Antioxidants.

[16] Gauthier M., Senhorinho G., Scott J. (2020). Microalgae under Environmental Stress as a Source of Antioxidants. Algal Res..

[17] Edelmann M., Aalto S., Chamlagain B., Kariluoto S., Piironen V. (2019). Riboflavin, Niacin, Folate and Vitamin B12 in Commercial Microalgae Powders. J. Food Compos. Anal..

[18] Del Mondo A., Smerilli A., Sané E., Sansone C., Brunet C. (2020). Challenging Microalgal Vitamins for Human Health. Microb. Cell Fact..

[19] Ovando C.A., Carvalho J.C.D., de Melo Pereira G.V., Jacques P., Soccol V.T., Soccol C.R. (2018). Functional Properties and Health Benefits of Bioactive Peptides Derived from Spirulina: A Review. Food Rev. Int..

[20] Çelekli A., Alslibi Z.A., Bozkurt H. (2019). Influence of Incorporated Spirulina Platensis on the Growth of Microflora and Physicochemical Properties of Ayran as a Functional Food. Algal Res..

[21] Pina-Pérez M.C., Brück W.M., Brück T., Beyrer M., Galanakis C.M. (2019). Microalgae as Healthy Ingredients for Functional Foods. The Role of Alternative and Innovative Food Ingredients and Products in Consumer Wellness.

[22] Abu-Ghosh S., Dubinsky Z., Verdelho V., Iluz D. (2021). Unconventional High-Value Products from Microalgae: A Review. Bioresour. Technol..

[23] Abdelkhalek N.K.M., Ghazy E.W., Abdel-Daim M.M. (2015). Pharmacodynamic Interaction of Spirulina Platensis and Deltamethrin in Freshwater Fish Nile Tilapia, *Oreochromis niloticus*: Impact on Lipid Peroxidation and Oxidative Stress. Environ. Sci. Pollut. Res..

[24] Abdel-Daim M.M., Dawood M.A., Elbadawy M., Aleya L., Alkahtani S. (2020). Spirulina Platensis Reduced Oxidative Damage Induced by Chlorpyrifos Toxicity in Nile Tilapia *(Oreochromis niloticus*). Animals.

[25] Vingiani G.M., De Luca P., Ianora A., Dobson A.D., Lauritano C. (2019). Microalgal Enzymes with Biotechnological Applications. Mar. Drugs.

[26] Elleuch J., Hadj Kacem F., Ben Amor F., Hadrich B., Michaud P., Fendri I., Abdelkafi S. (2021). Extracellular Neutral Protease from *Arthrospira platensis*: Production, Optimization and Partial Characterization. Int. J. Biol. Macromol..

[27] Brasil B.d.S.A.F., de Siqueira F.G., Salum T.F.C., Zanette C.M., Spier M.R. (2017). Microalgae and Cyanobacteria as Enzyme Biofactories. Algal Res..

[28] Sharifian S., Homaei A., Kim S.-K., Satari M. (2018). Production of Newfound Alkaline Phosphatases from Marine Organisms with Potential Functions and Industrial Applications. Process. Biochem..

[29] Chu Y.-H., Yu X.-X., Jin X., Wang Y.-T., Zhao D.-J., Zhang P., Sun G.-M., Zhang Y.-H. (2019). Purification and Characterization of Alkaline Phosphatase from Lactic Acid Bacteria. RSC Adv..

[30] Shao W., Ebaid R., El-Sheekh M., Abomohra A., Eladel H. (2019). Pharmaceutical Applications and Consequent Environmental Impacts of Spirulina (*Arthrospira*): An Overview. Grasas Aceites.

[31] Lafarga T., Fernández-Sevilla J.M., González-López C., Acién-Fernández F.G. (2020). Spirulina for the Food and Functional Food Industries. Food Res. Int..

[32] Almeida L.M.R., da Silva Cruz L.F., Machado B.A.S., Nunes I.L., Costa J.A.V., de Souza Ferreira E., Lemos P.V.F., Druzian J.I., de Souza C.O. (2021). Effect of the Addition of Spirulina Sp. Biomass on the Development and Characterization of Functional Food. Algal Res..

[33] Şahin O.I. (2020). Functional and Sensorial Properties of Cookies Enriched with Spirulina and Dunaliella Biomass. J. Food Sci. Technol..

[34] Casciano F., Nissen L., Gianotti A. (2021). Effect of Formulations and Fermentation Processes on Volatile Organic Compounds and Prebiotic Potential of Gluten-Free Bread Fortified by Spirulina (*Arthrospira platensis*). Food Funct..

[35] Padalino L., Lecce L., Chini Zittelli G., Lo Grieco A., Yorzillo G. (2017). Use of Spirulina to Enhance the Nutritional Value of Durum Wheat Spaghetti. Food Nutr. J..

[36] Bosnea L., Terpou A., Pappa E., Kondyli E., Mataragas M., Markou G., Katsaros G. (2020). Incorporation of Spirulina Platensis on Traditional Greek Soft Cheese with Respect to Its Nutritional and Sensory Perspectives. Proceedings.

[37] Galafat A., Vizcaíno A.J., Sáez M.I., Martínez T.F., Jérez-Cepa I., Mancera J.M., Alarcón F.J. (2020). Evaluation of *Arthrospira* sp. Enzyme Hydrolysate as Dietary Additive in Gilthead Seabream (*Sparus aurata*) Juveniles. J. Appl. Phycol..

[38] Khan S., Mobashar M., Mahsood F.K., Javaid S., Abdel-Wareth A., Ammanullah H., Mahmood A. (2020). Spirulina Inclusion Levels in a Broiler Ration: Evaluation of Growth Performance, Gut Integrity, and Immunity. Trop. Anim. Health Prod..

[39] Martins C.F., Pestana Assuncao J., Ribeiro Santos D.M., Madeira M.S.M.d.S., Alfaia C.M.R.P.M., Lopes P.A.A.B., Coelho D.F.M., Cardoso Lemos J.P., de Almeida A.M., Mestre Prates J.A. (2021). Effect of Dietary Inclusion of Spirulina on Production Performance, Nutrient Digestibility and Meat Quality Traits in Post-Weaning Piglets. J. Anim. Physiol. Anim. Nutr..

[40] Markou G. (2012). Alteration of the Biomass Composition of *Arthrospira* (Spirulina) *platensis* under Various Amounts of Limited Phosphorus. Bioresour. Technol..

[41] Quisel J.D., Wykoff D.D., Grossman A.R. (1996). Biochemical Characterization of the Extracellular Phosphatases Produced by Phosphorus-Deprived *Chlamydomonas reinhardtii*. Plant Physiol..

[42] Golotin V., Balabanova L., Likhatskaya G., Rasskazov V. (2015). Recombinant Production and Characterization of a Highly Active Alkaline Phosphatase from Marine Bacterium *Cobetia marina*. Mar. Biotechnol..

[43] Droop M.R. (1968). Vitamin B12 and Marine Ecology. IV. The Kinetics of Uptake, Growth and Inhibition in *Monochrysis lutheri*. J. Mar. Biol. Assoc..

[44] Carvalho A., Silva S., Baptista J., Malcata F. (2011). Light Requirements in Microalgal Photobioreactors: An Overview of Biophotonic Aspects. Appl. Microbiol. Biotechnol..

[45] Adams C., Godfrey V., Wahlen B., Seefeldt L., Bugbee B. (2013). Understanding Precision Nitrogen Stress to Optimize the Growth and Lipid Content Tradeoff in Oleaginous Green Microalgae. Bioresour. Technol..

[46] Ghyoot C., Gypens N., Flynn K.J., Lancelot C. (2015). Modelling Alkaline Phosphatase Activity in Microalgae under Orthophosphate Limitation: The Case of *Phaeocystis globosa*. J. Plankton Res..

[47] Whitton B.A., Al-Shehri A.M., Ellwood N.T., Turner B.L. (2005). Ecological Aspects of Phosphatase Activity in Cyanobacteria, Eukaryotic Algae and Bryophytes. Organic Phosphorus in the Environment.

[48] Girault M., Siano R., Labry C., Latimier M., Jauzein C., Beneyton T., Buisson L., Del Amo Y., Baret J.-C. (2021). Variable Inter and Intraspecies Alkaline Phosphatase Activity within Single Cells of Revived Dinoflagellates. ISME J..

[49] Mo Y., Ou L., Lin L., Huang B. (2020). Temporal and Spatial Variations of Alkaline Phosphatase Activity Related to Phosphorus Status of Phytoplankton in the East China Sea. Sci. Total Environ..

[50] Pandey S., Banik R. (2010). Optimization of Process Parameters for Alkaline Phosphatase Production by *Bacillus licheniformis* using Response Surface Methodology. Int. J. Agric. Technol..

[51] Zhou Y., Lu Z., Wang X., Selvaraj J.N., Zhang G. (2018). Genetic Engineering Modification and Fermentation Optimization for Extracellular Production of Recombinant Proteins using *Escherichia coli*. Appl. Microbiol. Biotechnol..

[52] Sebastian M., Ammerman J.W. (2009). The Alkaline Phosphatase PhoX is More Widely Distributed in Marine Bacteria Than the Classical PhoA. ISME J..

[53] Murphy J.E., Kantrowitz E.R. (1994). Why are Mammalian Alkaline Phosphatases Much More Active than Bacterial Alkaline Phosphatases?. Mol. Microbiol..

[54] Thengodkar R.R.M., Sivakami S. (2010). Degradation of Chlorpyrifos by an Alkaline Phosphatase from the Cyanobacterium Spirulina Platensis. Biodegradation.

[55] Asencio A.D., Morte A., García-Carmona F., Pérez-Gilabert M. (2012). Partial Purification and Characterization of a Calcium-Dependent Alkaline Phosphatase from the Cyanobacterium *Arthrospira platensis*. J. Phycol..

[56] Singh S., Singh S., Pandey V., Mishra A. (2006). Factors Modulating Alkaline Phosphatase Activity in the Diazotrophic Rice-field Cyanobacterium, *Anabaena oryzae*. World J. Microbiol. Biotechnol..

[57] Upadhyay L.S.B., Verma N. (2015). A Three Step Approach for the Purification of Alkaline Phosphatase from Non-Pasteurized Milk. J. Food Sci. Technol..

[58] Plocke D.J., Levinthal C., Vallee B.L. (1962). Alkaline Phosphatase of *Escherichia coli*: A Zinc Metalloenzyme. Biochemistry.

[59] McComb R.B., Bowers G.N., Posen S. (2013). Alkaline Phosphatase.

[60] Lopez A., Pique M., Boatella J., Parcerisa J., Romero A., Ferrá A., Garcí J. (1997). Influence of Drying Conditions on the Hazelnut Quality. II. Enzymatic Activity. Dry Technol..

